# Distributional Cost-Effectiveness Analysis Comes of Age

**DOI:** 10.1016/j.jval.2020.10.001

**Published:** 2021-01

**Authors:** Richard Cookson, Susan Griffin, Ole F. Norheim, Anthony J. Culyer, Kalipso Chalkidou

**Affiliations:** 1 Centre for Health Economics, University of York, York, England, UK; 2 Department of Global Public Health and Primary Care, University of Bergen, Norway; 3 Faculty of Medicine, School of Public Health, Imperial College London, London, England, UK

## Introduction

Distributional cost-effectiveness analysis (DCEA) provides information about the equity impacts of health technologies and programs and the trade-offs that sometimes arise between equity and efficiency. This field has now come of age with a growing applied literature,^1^ new training resources,^2^ and a formal professional network: a special interest group on equity-informative economic evaluation within the International Health Economics Association.^3^

A systematic review published in this issue of *Value in Health*^1^ found 54 peer-reviewed studies published to date, mostly after 2015, relating to diverse disease categories, intervention types, and populations and using various equity criteria, with socioeconomic status and race/ethnicity being the most frequent. Most reviewed studies (78%) found a favorable equity impact of the health program under investigation, and only 6% found an unfavorable equity impact.

This may be a sign of publication bias, whereby equity analysis is more likely to be conducted in cases where a favorable impact is anticipated. It may also indicate a tendency to focus on the favorable distribution of benefits rather than the unfavorable distribution of burdens owing to opportunity costs. Both issues require attention, because decision makers need to know when equity impacts are unfavorable and they need a full picture of equity impacts including who bears the largest burdens of opportunity cost as well as who gains the largest benefits.

## Background

Concern about health inequalities has been given further impetus in recent years by so-called “deaths of despair” from suicide, drug overdose, and alcoholic liver disease^4^ and, more recently, inequalities in coronavirus infection and mortality rates related to ethnicity and socioeconomic status.^5^ A new book^2^ now provides practical methods for analyzing the expected impacts of healthcare and public health programs on inequalities between advantaged and disadvantaged groups in health, health service use, and the financial hardship resulting from health service use (eg, owing to out-of-pocket costs).^6^, ^7^, ^8^, ^9^ These methods supplement conventional cost-effectiveness data with distributional breakdowns and equity weighting analyses based on social variables such as socioeconomic status, ethnicity, and geographical location and disease categories like disability and severity of illness.^8^^,^^10^

Effectiveness studies (trials, quasi-experiments, and evidence synthesis thereof) can sometimes give partial information on equity impacts, for example, through subgroup analysis or trials in disadvantaged populations.^11^ Nevertheless, effectiveness studies usually fail to address equity issues of interest to decision makers:

• the distribution of the health opportunity costs of cost-increasing programs
• distributional impacts within the general population
• impacts on inequality in health outcomes beyond the trial follow-up period
• sizes of health inequality impacts compared with other programs
• trade-offs between equity and efficiency objectives.

## Distributional Cost-Effectiveness Analysis

The general term we favor for this form of evaluation is *distributional cost-effectiveness analysis* (DCEA).^2^ We use DCEA as an umbrella term for any study that provides information about equity in the distribution of costs and effects as well as value for money. This approach uses additional evidence and modeling to evaluate equity impacts and trade-offs and can be useful whenever a decision is expected to have different consequences for different people:

• costly new health technologies (eg, whether to fund new a treatment or vaccination, at what price, and for which population subgroups)
• coverage in healthcare benefit packages (eg, whether to cover diabetes in a public health insurance plan and, if so, which treatments and with what copayments)
• new health service delivery infrastructure (eg, whether to invest in a community health worker program, how to select target areas for new investments)
• public health (eg, whether to implement a sugar-sweetened beverage tax, how to evaluate health effects with effects on private household consumption and expenditure)

*Distributional Cost-Effectiveness Analysis: Quantifying Health Equity Impacts and Trade-Offs* is a guide for research commissioners, users, students, and analysts.^2^ The introductory chapters summarize how DCEA applies in various decision-making contexts and defines the concepts needed to read and critically appraise DCEA studies.

DCEA can explore the implications of giving special priority to improving the health of program recipients compared to nonrecipients.^12^^,^^13^ It can also analyze the distribution of health benefits and burdens (opportunity costs) within the general population by equity-relevant social variables (eg, socioeconomic status, geographical location, indigenous status, ethnicity, sex, age) and disease categories (eg, disease classification, severity of illness, proximity to death, rarity of condition).^14^ It can also evaluate distributional consequences for nonhealth outcomes, such as income or financial protection from out-of-pocket healthcare costs,^15^ and locate potential trade-offs between equity and efficiency objectives.^16^ Depending on the question at hand, additional modeling ranges from simple decision trees to sophisticated microsimulation.^17^

The distinctive aim is to extract new information about equity out of the analysis, rather than to incorporate value judgments about equity into it. DCEA is not about finding an algorithmic approach to replace context-specific deliberation with a universal equity formula. Rather, it can be used as an input into context-specific deliberation by decision makers and stakeholders. Standard cost-effectiveness studies inescapably make value judgments about equity—but implicitly. A common one is, for example, the value judgment that all health-adjusted life-years are equally valuable.^18^ DCEA makes these judgments explicit. It specifies the kinds of distributional consequences to expect and proposes measures. At a minimum, it can provide simple descriptive information.

## The Equity-Efficiency Impact Plane

The equity-efficiency impact plane is a way of visualizing the findings of a DCEA study.^19^ This enables thinking about trade-offs between efficiency and equity and can also help decision makers keep both objectives in sight and in balance.

**Figure 1:**
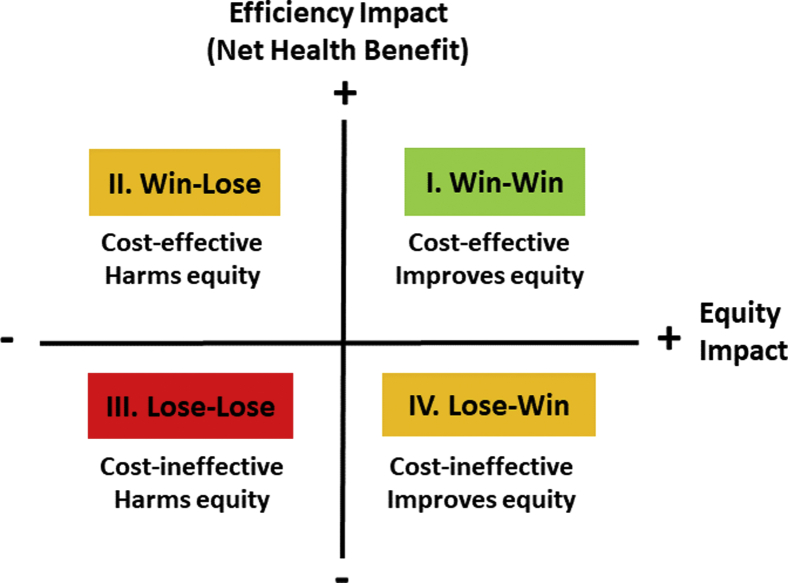
Equity-efficiency impact plane.

According to the standard net health benefit framework, a program is cost-effective if its health benefit is greater than its health opportunity cost.^20^ Nevertheless, if it harms equity then a cost-effective program might not be worth implementing. And if a cost-ineffective program improves equity, then it might be worth implementing. The equity-efficiency impact plane in Figure 1 sets out the 4 logical possibilities.

The origin of the equity-efficiency impact plane locates a comparator intervention. The vertical axis tells us whether an alternative option is better than the comparator in terms of efficiency, and the horizontal axis tells us whether it is better in terms of equity.

Equity impact can be measured in whatever units are of interest to the decision maker. In England and Ethiopia, for example, researchers have used a reduction in an index of inequality in deprivation-related inequality in health-adjusted life expectancy.^21^^,^^22^ Nevertheless, other measures will suit different policy contexts—including objective health measures such as infant mortality and simple equity metrics such as the gap between the best and worst off.

A policy that falls in the northeast win-win quadrant improves both total health and health equity, and one that falls in the southwest lose-lose quadrant harms both. In low- and middle-income countries, vaccination programs (eg, rotavirus immunization)^23^ and infectious disease control programs (eg, tuberculosis)^24^ often fall into the win-win quadrant, because they typically deliver large health gains per unit cost and disproportionately benefit socially disadvantaged groups. By contrast, investments in high-cost end-of-life treatments may fall into the lose-lose quadrant of being neither cost-effective nor likely to reduce social inequality in health. Coverage of interventions in the lose-lose quadrant will rely on other ethical and political arguments of value.^25^

Equity and efficiency impacts may also be opposed. In the northwest win-lose quadrant, the option is good for total health but bad for equity, and in the southeast lose-win quadrant, the option is bad for total health but good for equity. This can happen, for example, when socially disadvantaged groups gain less than advantaged groups from a decision to fund a medical technology, due perhaps to barriers to access, adherence, and long-term recovery, and additional investment in delivery infrastructure and follow-up care would be needed to facilitate equal access, adherence, and long-term recovery.

## Quick and Dirty Approaches

Simplified aggregate approaches are also available, when there is not enough time or resource to conduct a full DCEA study. These combine aggregate outputs from standard cost-effectiveness analysis with information about distributions of a relevant disease or risk factor and utilization of a relevant category of care.^22^^,^^26^ This can provide useful prima facie information about equity impact, even though there is no detailed modeling.

## Equity-Efficiency Trade-Offs

In the win-lose and lose-win cases, equity trade-off analysis is required to discover which policy is better overall. This analysis can be done informally by making intuitive judgments. It can also be done formally as the following examples show:

• inequality indices that assign numerical values to equity impacts
• dominance tests based on simple underlying principles or axioms of equity
• indirect equity weighting (ie, quantifying an overall social value for each policy by using a social welfare function with an equity parameter, which reflects degree of concern for the worse off and indirectly implies equity weights)
• direct equity weighting (ie, formally comparing the policy options by setting direct equity weights on health benefits for special groups)

The methods chapters of the book^2^ contain step-by-step instructions on how to conduct an equity-informative study, with accompanying spreadsheet training exercises^27^ and an online tool^28^ for summarizing equity impacts and trade-offs. There are also online resources for those interested more specifically in equity-informative economic evaluation in low- and middle-income countries.^29^^,^^30^ Updates on training materials and courses are available via the International Health Economics Association Special Interest Group on equity-informative economic evaluation.^3^

## Conclusion

*Distributional Cost-Effectiveness Analysis: Quantifying Health Equity Impacts and Trade-Offs* and its training materials, together with the recent review*,* should stimulate studies that combine efficiency and equity in all countries, whatever their stage of development, where equity in health and healthcare is of concern, or where aspirations to universal health coverage are an imperative. These developments should also spur theorists and practitioners alike to develop further techniques and create better data for decisions and, of course, better decisions.

## Article and Author Information

**Accepted for Publication:** October 6, 2020

**Published Online:** November 7, 2020

doi: https://doi.org/10.1016/j.jval.2020.10.001
